# Comparison of Tunable Diode Laser Absorption Spectroscopy and Isothermal Micro-calorimetry for Non-invasive Detection of Microbial Growth in Media Fills

**DOI:** 10.1038/srep27894

**Published:** 2016-06-10

**Authors:** David Brueckner, David Roesti, Ulrich Georg Zuber, Rainer Schmidt, Stefan Kraehenbuehl, Gernot Bonkat, Olivier Braissant

**Affiliations:** 1 Center of Biomechanics & Biocalorimetry, University Basel, Gewerbestr. 14, CH-4123 Allschwil, Switzerland; 2 F. Hoffmann – La Roche, Ltd., Sterile Drug Product Manufacturing, Wurmisweg, CH-4303 Kaiseraugst, Switzerland; 3 Novartis Pharma Stein AG, CH-4332 Stein, Switzerland; 4 Clinical Pharmacology & Toxicology, Universitätsspital Basel, Markgräflerhof, Hebelstrasse 2, CH-4031 Basel, Switzerland; 5 Department of Urology, Universitätsspital Basel, Spitalstrasse 21, CH-4031 Basel, Switzerland

## Abstract

Two methods were investigated for non-invasive microbial growth-detection in intact glass vials as possible techniques for automated inspection of media-filled units. Tunable diode laser absorption spectroscopy (TDLAS) was used to determine microbially induced changes in O_2_ and CO_2_ concentrations within the vial headspaces. Isothermal microcalorimetry (IMC) allowed the detection of metabolic heat production. *Bacillus subtilis* and *Streptococcus salivarius* were chosen as test organisms. Parameters as robustness, sensitivity, comparability and time to detection (TtD) were evaluated to assess method adequacy. Both methods robustly detected growth of the tested microorganisms within less than 76 hours using an initial inoculum of <10CFU. TDLA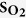 turned out to be less sensitive than TDLA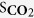 and IMC, as some false negative results were observed. Compared to the visual media-fill examination of spiked samples, the investigated techniques were slightly slower regarding TtD. Although IMC showed shorter TtD than TDLAS the latter is proposed for automating the media-fill inspection, as larger throughput can be achieved. For routine use either TDLA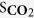 or a combination of TDLA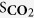 and TDLA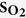 should be considered. IMC may be helpful for replacing the sterility assessment of commercial drug products before release.

Aseptic processing is used to manufacture products that are intended to be sterile and ultimately injected to patients. The process is periodically simulated by filling microbial culture media (tryptic soy broth – TSB) instead of the drug product. This simulation is designated as a “media fill” and ensures the reliability and repeatability of aseptic processing^1^,^2^ as even in pharmaceutical clean room environment a diverse microbial community can be found^3^. In conventional media fill procedures, intact media-filled vials are incubated for 7 days at 20–25 °C and another 7 days at 30–35 °C^4^,^5^. After 7 and 14 days, microbial contamination of media filled units is assessed by an operator through a visual inspection (VI), searching for an increased media turbidity as well as modifications in the aspect of media. However, VI is limited by several drawbacks including high and repetitive workload, weak data integrity, assumptions on media turbidity that is prone to human error, and inefficient data collection^6^,^7^. Although these drawbacks ensure patient safety and good manufacturing practices (GMP), they are associated with costs. Also the long turnover time required by incubation and examination of media fills leads to business risk regarding the sterility requirements if manufacturing has already resumed. Therefore, the establishment of new, non-invasive, more efficient, objective and faster methods replacing the conventional visual media fill inspection is needed. In this paper tunable diode laser absorption spectroscopy (TDLAS), measuring gas concentrations in vial headspaces, and isothermal micro-calorimetry (IMC), measuring metabolic heat production of microorganisms, are investigated as alternative methodologies.

The development of spectroscopic measurement techniques in the near- and mid-infrared spectral region^8^,^9^,^10^ has led to their extensive use in the pharmaceutical industry for e.g., the determination of trapped gas inside vials, the analysis of porosity in tablets and the control of freeze-drying processes^11^,^12^,^13^. Considering parenteral drug production, contamination by microbes from the environment is a constant threat during aseptic filling^3^. These microbes are mostly aerobic and thus the standard media fill incubation is performed in aerobic conditions. Microorganisms using respiration as metabolic pathway consume oxygen and release carbon-dioxide (see equation 1). Non-invasive spectroscopic measurement techniques, such as TDLAS, can be used to detect growth related changes of O_2_ and CO_2_ concentrations in headspaces of sealed vials^14^. This provides insights on microbial contamination of media filled units. However, microbial fermentation can also occur and result in other end products such as lactic acid and alcohols (see equations 2 and 3). Nevertheless, many of these fermenters are not limited to their primary pathways and may therefore remain detectable by TDLAS. Still for some of those fermenters differences in cultivation medium composition (i.e., from different manufacturer, or different batches) might lead to use of metabolic pathway with little to no O_2_ consumption and CO_2_ production.


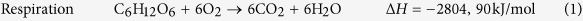



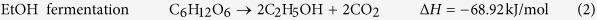



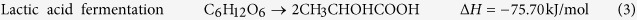


In addition, metabolic reactions resulting from microbial proliferation emit heat which can be quantified continuously and non-invasively by isothermal micro-calorimetry (IMC). IMC therefore detects enthalpy changes associated with the cell metabolism (see ΔH of equations 1–3) and has been used already to monitor microbial growth^15^,^16^,^17^,^18^,^19^.

Here TDLAS and IMC are evaluated and compared as possible alternatives to current inspection procedures for parenteral drugs, aiming at a high-throughput substitution of the visual media fill inspection. Parameters such as robustness, sensitivity (i.e., the proportion of false negative), sensibility (i.e., the instrumental detection limit in our conditions), time to detection and comparability are investigated for each method, using the fast-growing, metabolically versatile *Bacillus subtilis* and the lactic acid fermenter *Streptococcus salivarius* as model contaminants. These organisms have been chosen as they represent extremes in terms of O_2_ depletion and CO_2_ production found in a preliminary screening. Growth measurements obtained using the different methods are compared using the Gompertz growth model^20^.

## Results

**Figure 1:**
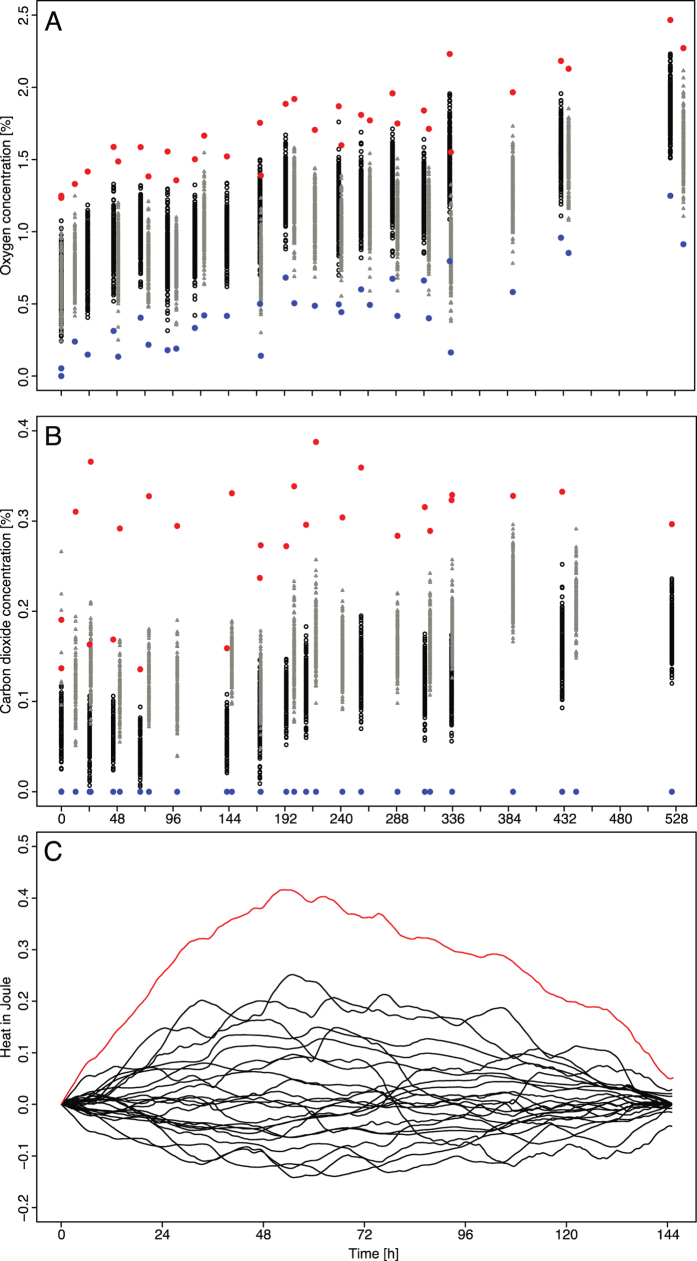
Plot (**A**) describes the inverted O_2_ baseline development. It was determined by calculating the difference of O_2max_ − O_2_ for all measurement series. The red and blue dots describe the upper and lower 4σ confidence intervals. Plot (**B**) describes the CO_2_ baseline development. The red dots show the upper 4σ confidence intervals whereby the blue dots stand for the physical limits being equal to zero. The CO_2_ and O_2_ data is based on an old (grey) and a new (black) TSB lot being measured during at least 14 days. Plot (**C**) shows the heat emission during 145 hours of 24 blank TSB samples of three TSB lots differing in age. The super-ordinated red curve shows the 4σ confidence interval. Its maximum peak defines the threshold parameter for IMC.

To determine microbial growth of *Streptococcus salivarius* and *Bacillus subtilis* by TDLAS and IMC a threshold was defined by a 4σ confidence interval defined on unicolutated samples. Repeated measurements of sterile TSB samples on heat production and headspace change in CO_2_ and O_2_ concentration provided data on the biochemical processes occurring in the medium, influencing heat flow and gas levels (Fig. 1 - section 2.1).

**Figure 2:**
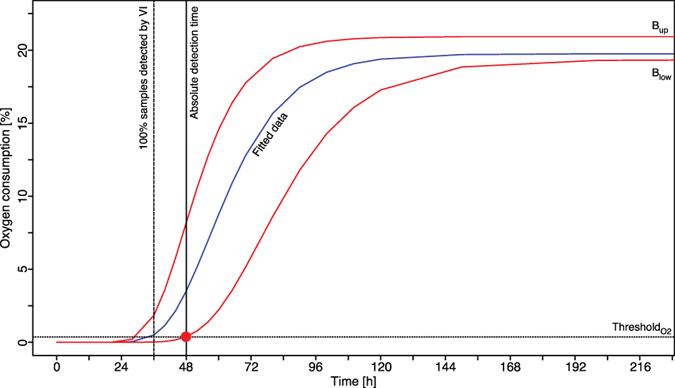
Example of an inverse O_2_ growth profile of *Bacillus subtilis*. The inverted profile was determined by calculating the difference of O_2max_ − O_2_ out of a collection of 60 inoculated vials. The red s-shaped curves illustrate upper (B_up_) and lower (B_low_) boundaries of growth associated oxygen consumption. The individual blue profile is the averaged fit of the entire sample collection. The dashed vertical line illustrates the time needed to detect visually 100% of all inoculated samples. The solid vertical line defines the absolute TtD for *Bacillus subtilis* based on oxygen measurements. The red dot marks the intersection of the lower boundary and the threshold 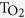.

**Figure 3:**
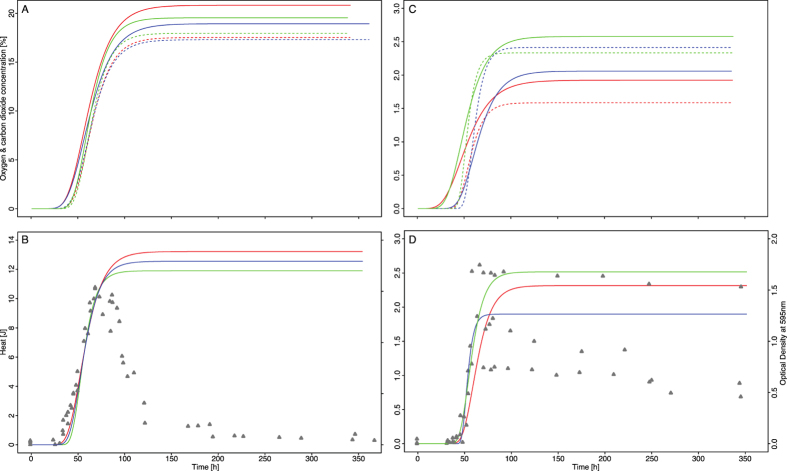
Fitted gas and thermogenic profiles together with raw data of OD_595_ measurements for *B. subtilis* and *S. salivarius*. Plots (**A**,**B**) illustrate plotted data for *Bacillus subtilis*, (**C**,**D**) plotted data for *Streptococcus salivarius*. The development of CO_2_ (dotted) and O_2_ (solid) profiles in fitted form are visualized in graph (**A**,**C**) for both organisms, whereby the color red describes the development of the first run, blue the second run and green the third run, including 20 samples each. Plot (**B**,**D**) visualize fitted heat (solid) of three runs with TSB differing in age and raw data of various OD_595_ measurements (Δ) over time. Considering the heat and gas profiles in more detail it becomes obvious that despite small deviations in profile development reproducibility and robustness is given for TDLAS and IMC measurements.

For inoculated samples, the intersection of fitted growth curves with the respective threshold defined the time to detection (TtD) (section 2.2). In turn, the distributions of TtD allowed making assumptions on method robustness, sensitivity, sensibility and comparability to visual inspection. In addition, an absolute TtD was determined for each organisms and method. Therefore worst-case fitting parameters of the Gompertz model (i.e., maximal λ (i.e., the longest lag phase) with smallest μ (lowest growth rate) and lowest X_max_ (minimum gas concentration change or heat produced)) were combined in this model to create a worst-case growth curve. The intersection of this worst-case growth curve with the respective threshold determined absolute TtD (Fig. 2). In parallel to each TDLAS and IMC measurement vials were inspected visually on the increase in turbidity. In addition, optical density was measured to allow a comparison of this commonly used measure with TDLAS and IMC profiles (Table 1, Fig. 3).

### Thresholds for gases concentration ( 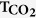 , 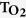 ), heat production (T_H_) and optical density (T_OD_)

In sterile TSB filled vials slight variations of oxygen and carbon-dioxide concentration were observed. Overall 82 vials out of 84 (2 contaminated samples were rejected) filled with two TSB lots differing in age were used to investigate these fluctuations in vial headspace. From the 14′645 TDLAS measurements obtained, 2′460 data points (16.8%) were omitted due to non-normal distribution of the data series, hindering the use of the 4σ approach. Another 2′025 (13.8%) points below detection limit were removed. Based on the remaining data (69.4%) 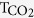 and 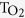 were determined. To define the IMC threshold for growth detection 24 vials filled with three TSB lots differing in age were continuously measured. No vials or measurement points were rejected.

After 7 days 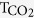 was defined at 0.366% and 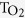 at 1.754%. T_H_ was defined at 0.416J and T_OD_ at 0.02 (Fig. 1).

### Growth Profile and Parameter Analysis

The two microorganisms tested were detected by all methods used with robust and reproducible results across all the independent experimental runs (Fig. 3). However, TtD and number of false negative results varied slightly (Table 1). Lag phase duration, growth rate and maximal concentration/heat/biomass reached could be calculated for all organisms investigated and methods used. *Bacillus subtilis* showed higher reproducibility in λ and μ and reached higher changes in gas concentration and heat production compared to *S. salivarius* (Table 1). Differences in heat production and gas concentration patterns between the 2 microorganisms is mainly attributed to the differences in *S. salivarius* and *B. subtilis* metabolism. Three TDLAS *S. salivarius* vials were not included due to secondary contamination.

TtD, the intersection between threshold and curve considered, for single curves of TDLA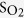, TDLA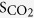, OD_595_ and IMC were determined. TDLAS, OD_595_ and IMC measurements detected all *B. subtilis* inoculated replicates between 28.3 h and 57.0 h. *S. salivarius* samples became positive between 40.5 h and 70.7 h. However, 10% of the *S. salivarius* oxygen profiles did not reach the threshold, meaning that less than 1.754% O_2_ was consumed after 7 days (Table 1). This emphasizes that the sensitivity of TDLA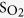 remained rather high as values were 100% and 90% for *B. subtilis* and *S. salivarius* respectively. Also, this supports the use of *S. salivarius* as worst-case scenario microbe.

Absolute TtD was modeled by using worst case parameters obtained for each organism with TDLA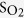, TDLA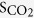 and IMC data. This time point was defined by the intersection of B_low_ (computed using largest λ, minimal μ and smallest X_max_ – see section 5.4) with the respective threshold and was comprised between 45.9 and 76 hours (Table 1). The absolute TtD values showed that IMC outperformed TDLAS but that VI remained the fastest detection method. TtD for OD_595_ (used only for comparison purposes) was comparable to visual TtD and in most cases faster. However VI is performed only after day 7.

## Discussion

TDLAS and IMC were evaluated for non-invasive microbial growth-detection in intact glass vials aiming at a rapid and automated inspection of media-filled units. Both methods proved to be technologies capable of detecting growth of *Bacillus subtilis* and *Streptococcus salivarius* in sealed vials. The results were robust and showed good reproducibility. In addition, TDLAS and IMC are comparable to the conventional visual media fill inspection performed after 7 days in terms of detection speed. However, 10% of all *S. salivarius* vials did not reach the set threshold (false negatives). This phenomenon was mainly linked with the auto-oxidative characteristic of TSB which affected the threshold chosen and resulted in a 4.8 times higher oxygen threshold compared to CO_2_. Furthermore, the metabolism from *S. salivarius* led to relatively low O_2_ consumption and CO_2_ production, which might indicate the use of alternative pathways. As a consequence, oxygen depletion in vial headspaces was insufficient to reach the threshold. Considering reproducibility and robustness, different sources of variations exist. The origin of variations between runs of the same microorganism could be due to the different TSB lot used as this medium is not defined and variations in composition exist between batches and between manufacturers. The low CFU count used for the inoculation might be an additional source of errors as very little variation in the CFU number or the viability of the cells might have affected their growth. Finally the tendency of streptococci for self aggregation might also have influenced the reproducibility for this organism as inoculum size might have varied.

The sensibility for each method with respect to detection speed of microbial growth was clearly demonstrated (Fig. 3). With an inoculum <10 CFU per vial IMC outperformed TDLAS in terms of TtD. Still, it might be even optimized further. The calorimeter used in our experiment is an instrument of the mid-range performance class, originally designed for studying cement curing^21^. The 2 ml vial format did not perfectly fit in the calorimeter’s sample holder and results with even higher quality would have been obtained with samples of higher heat capacity and conductivity (i.e., 15–20 ml vials). The setup with a 2 ml vial inserted in a 20 ml plastic vial fitting into the IMC sample receiver probably resulted in additional measurement noise (i.e., increased limit of detection). Overall, there are many possibilities to further improve the IMC setup and to decrease the threshold thus resulting in lower TtD. The TDLAS setup had also some drawbacks. The short diameter of the 2 ml format results in a shorter measurement path than compared to containers of larger size. This leads to analytical fluctuations^12^ and causes additional measurement errors. An improvement of measurement precision could be reached by using containers with larger diameters. However, the use of larger formats is also linked with bigger headspaces that might require more O_2_ to be consumed or CO_2_ to be produced to reach a similar gas concentration level. Thus, in contrast to IMC that measures heat produced in the whole vial independently of the vial format, TDLAS might be less appropriate for larger formats when fermenting microorganisms are considered as the larger filling volume is compensated by a larger headspace.

From an operational perspective, the visual inspection of a media fill assessment is comparable to the alternative methods investigated if growth detection occurs before the first inspection step. Indeed, first visual inspection activities are performed after 7 days (168 hours), meaning that despite variations in TtD all methods would have detected growth of *B. subtilis* or *S. salivarius* before the standard visual inspection.

However, it was observed that within the first 7 days OD_595_ delivered best performance in detection speed whereby IMC and TDLAS were slower than the visual inspection. Despite OD_595_’s advantage in TtD its use in replacing the visual read-out of all vials (ca 10,000 per media fill) is not an option as sediments of growing microorganisms can fall to the vial bottom or form micro colonies. In this context, a shaking or vortexing step would be difficult to implement in an automated production line. Alternatively, in some cases such measurements might be impaired by autolysis as well (Fig. 3B,D). This would promote the risk for obtaining false negative results and complicate automated vial inspection. In addition, further applications of optical density measurements to poorly soluble drugs, provided as suspension or dispersion, is not possible due to the optical characteristic of such products.

Before a substitution of visual media fill read-out can be realized by either IMC or TDLAS, additional experiments are necessary to create a reliable decision base. Besides evaluating the impact on precision by using larger vial formats such studies should incorporate a specificity analysis by looking at a broader range of organisms with different metabolic pathways that are known contaminants in pharmaceutical drug manufacturing. Depending on the method and format the threshold will require a reassessment to provide an optimal specificity combined with a short TtD. Furthermore, microorganisms in drug manufacturing facilities are likely exposed to a lack of nutrition, heat shocks, hyperacidity, remnants of antibiotics, disinfectants, or other external influences. This results in cellular stress and in an extended lag phase, having a direct impact on TtD. Within this context it might be valuable to also include a growth development analysis of stressed organisms in future studies. Vial non-integrity is another issue that needs to be investigated as an exchange of gases between headspace and atmosphere could considerably impact the results. Indeed the false negative rate and TtD might be increased by impaired vial integrity.

When dealing with such technologies it is crucial to know what can be achieved, taking into account advantages and drawbacks of each method individually (Table 2).

Besides IMC’s benefit of short TtD, the measurements are easy to handle and provide continuous real-time data^15^. Measuring heat-flow continuously can be an advantage as it allows getting some additional insights on metabolism of the potential contaminants, but also be a drawback since the throughput is limited to the number of measurement slots. In addition, metabolic heat emission is a temporary limited process, meaning that once growth terminates IMC cannot retrospectively assess bacterial contamination^22^. Growth of *B. subtilis* and *S. salivarius* stops after less than 150 hours (6.25 days), whereby the first inspection is planned at the 7^th^ day of incubation. This calls for a method where metabolic activities remain detectable independently of the moment of inspection. O_2_ and CO_2_ changes in intact vial headspaces remain rather constant after bacterial growth took place. Because of this and the characteristic of enabling a high-throughput read-out, the individual TDLA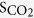 system (or in combination with TDLA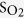) is recommended here as the method of choice for automated inspection of media fills. Nevertheless, IMC remains of interest in areas where continuous measurements are demanded with lower throughput requirements, e.g. sterility control of commercial drug products before release. Finally the combination of IMC and TDLAS for CO_2_ and O_2_ measurement allows to perform metabolic studies focusing on different pathways or microorganisms in which this pathways would have been altered by mutagenesis. Such studies are referred to as calorespirometric studies and could be of much use oustide the field of sterility testing for example in assessing product formation^23^,^24^,^25^.

## Conclusions

The non-invasive application together with an accurate detection of bacterial growth render IMC and TDLAS as valuable tools for inspecting media-filled units and potentially commercial drug product as well. TDLA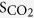 and IMC were capable to detect growth of *B. subtilis* and *S. salivarius* reliably within much less than 7 days. False negative results were observed for TDLA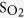 which makes its single use without parallel TDLA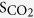 measurements questionable.

Although the verification of microbial growth by human visual inspection remained faster than TDLAS and IMC, its replacement stays a beneficial intention as augmented inspection objectivity, higher-throughput via automation, increased data integrity and efficient data collection can be realized. Due to the fact that automated vial handling for IMC is more difficult to achieve and less advanced than for TDLAS, the latter technique is suggested as substitution for visual media fill inspection despite IMC shorter TtD for growth detection. More potential is attributed to IMC in replacing the sterility assessment of commercial drug products before release. Nevertheless, further studies covering a wider range of microorganisms and formats are required to decide on whether or not IMC and/or TDLAS are appropriate method for a safer and more efficient sterility assessment procedures.

## Materials and Methods

### Microorganisms and culture conditions

*Bacillus* species are of obligate aerobe or facultative anaerobe, sporulating, gram-positive, rod-shaped nature and, appearing frequently in pharmaceutical environments^3^. They are often used in validation studies of microbial methods. In our study, *B. subtilis* (ATCC 6633) was obtained as bio balls^®^ (Thermo Scientific) validated to a cell count of <100 CFU and was routinely maintained aerobically at 30 °C on tryptic soy agar (TSA).

*Streptococcus salivarius* (ATCC 7073; DSM 20560) is a facultative-anaerobe, gram-positive, catalase- and oxidase-negative bacteria that belongs to the group of lactic acid fermenters. Indeed it produces mostly lactate from glucose, therefore affecting minimally CO_2_ and O_2_ concentrations. The organism was provided by DSMZ as cryo-culture and maintained aerobically at 37 °C on TSA.

### Preparation of experimental containers and bacterial suspensions

For all experiments 2 ml transparent, sterile tubed glass vials (Schott AG, Germany) were used. After manual filling of 1 ml TSB under aseptic conditions, containers were stoppered and capped with sterilized material and stored at 20–25 °C. As headspace gas concentrations are known to vary over time due to auto-oxidative processes in TSB^14^, each container was flushed for six seconds with sterile air at a flow rate of ~0.25 l/min before inoculation. This “headspace flushing” was intended to simulate the conditions prevailing in newly produced units.

Inoculation through the self-sealing rubber stopper was performed using a syringe filled with 0.1 ml bacterial suspension containing <100 CFU/ml (i.e., <10 CFU per vial). Suspensions including *B. subtilis* were prepared by dissolving a bio ball^®^ in Remel buffer, followed by diluting this suspension 1:10 times. *S. salivarius* was cultured twice for 48 h at 37 °C in TSB. The second culture was diluted by a factor of 1:10^−6^ in buffered sodium chloride-peptone bouillon and, with the addition of 15% glycerin, stored at −83 °C. The appropriate cell count for 0.1 ml suspension was determined by standard dilution series on agar plates.

### Experimental Design

#### Devices and Settings

Single pass bench-top TDLA Spectrometers (Lighthouse Instruments, Charlottesville, VA) were used. O_2_ and CO_2_ concentrations were determined at 762 nm and 2000 nm respectively. Before each experiment the devices were preheated for at least 30 minutes. A two-point calibration with certified standards (0%, 20%) provided by the manufacturer was performed before each measurement series. The measurement series were initiated after the measurement cap has been flooded with nitrogen (4 l/min) and the repeated analysis of standards with known concentration (4% and 8% respectively) was within specification limits (i.e., ±0.25% deviation). Standards used were made of the same glass as the experimental containers to keep the signal to noise ratio minimal. In parallel to each TDLAS measurement (lasting 5 seconds), a spectrophotometer (Bio Photometer plus, Eppendorf) was used to measure the optical density at 595 nm (OD_595_). OD_595_ data was gathered to allow comparison of this commonly used measure with TDLAS and IMC.

As initial experiments with TDLAS showed that the exponential growth phase of the organisms investigated had already stopped within less than hundred hours, it was decided to perform IMC experiments at 20°–25 °C only. For that, an eight-channel TamAir calorimeter (Waters/TA, Delaware, USA) was used. Once the eight samples were placed in the calorimeter, heat produced was recorded by the thermoelectric module placed between the samples and the heat-sink^15^. Reference vials used were identical to the experimental containers, but filled with sterile water thus providing an inert reference of similar heat capacity and conductivity. All 2 ml containers were additionally placed in a 20 ml plastic vial fitting the IMC sample receiver.

#### Baseline and Threshold Determination

To determine the threshold for microbial growth detection, sterile TSB samples were repeatedly measured for their heat production and headspace change in CO_2_ and O_2_ concentration. For TDLAS two sets of 42 vials were filled with new (0 days) and old (56 days) TSB. Their CO_2_ and O_2_ concentration was measured every 24 hours for at least 14 days. Each vial was analyzed 5 times to account for measurement variations^26^. The thresholds were defined after 7 and 14 days in order to be consistent with established inspection intervals.

To define the IMC threshold three sets of 8 vials containing TSB (3 up to 135 days in age) were measured continuously during 145 hours. This was considered as a sufficient time as the heat flow of all inoculated samples turned back to baseline after less than 100 hours.

For the definition of the OD_595_ threshold 100 measurements were performed with sterile TSB, filled in a cuvette.

#### Detection of bacterial growth by the different methods

For TDLAS measurements, three sets of TSB filled vials were prepared for both organisms. Each set included 20 inoculated vials and TSB lots varying from 1 day to 31 days in age. To reproduce the conditions of a real media fill, containers were incubated at 20–25 °C during 7 days and another 7 days at 30–35 °C. Before each measurement, vials were taken out of the incubator and were inspected visually by a qualified person for increased turbidity. After the 5 seconds lasting TDLAS measurement the containers were rapidly returned to the incubator. Measurements were taken every four hours during the growth phase.

Optical density (OD_595_) measurements were performed in parallel to the TDLAS measurements with vials from an additional set prepared identically. Their content was transferred into transparent plastic cuvettes and then measured on absorption at 595 nm. Blank samples were determined in TSB.

IMC experiments used three sets of TSB filled vials for both organisms. Each set included at least 6 inoculated vials. Measurements were taken continuously and resampled to achieve an effective sampling rate of 1 data point per minute.

All evaluated samples showing differences from typical and expected growth profiles of the organism under investigation, were sent to the PCR identification lab and omitted in case of non-conformity.

### Data Analysis

The data analysis described below was performed using the R statistical software^27^ in combination with the grofit package^28^.

#### Analyzing Threshold Data for CO_2_, O_2_, Heat, OD_595_, and VI

Each TDLAS measurement series (i.e., for each different time point) was checked on normality using the Shapiro-Wilk test. Non-normally distributed datasets, values equal to zero (i.e., below physical detection limit) and contaminated replicates were omitted from the analysis. The threshold parameters were estimated using a 4σ approach where the probability of receiving false positive results is around 0.006%. For the purpose of this study we choose to use 4σ instead of 3σ (commonly used in industry settings) to ensure safety of our results. The maximum value of mean plus four fold standard deviation was taken for the data gathered during the periods of 0 to 7 days and 7 to 14 days (see Fig. 1A,B) and considered as the threshold. The threshold parameter for emitted heat was defined by applying the 4σ approach to collected data and selecting the maximum of the 4σ confidence interval (Fig. 1C). The OD_595_ threshold was defined by the rounded 4σ confidence interval and the VI threshold by the perception of visible changes in turbidity of 100% of the inoculated samples.

#### Growth Profile Analysis and Data Transformation

Typical microbial growth appears in form of ascending sigmoidal curves (i.e., s-shaped curve). From a microbiology point of view, such curves can be described mathematically by 3 main parameters: lag phase duration (λ), exponential growth factor (μ) and maximal value reached (X_max_). Those parameters can easily be estimated for TDLAS, OD_595_ and IMC curves by using the Gompertz model, Oxygen concentration profiles are inverted growth curves by nature as they reflect oxygen consumption. To allow the use of the Gompertz model the oxygen profiles were converted to conventional s-shaped curves. For this O_2_ depletion was described as O_2max_ − O_2_. Heat flow [in μW (μJ/s)] over time is usually composed of one single (or several) peak(s). Therefore, the Gompertz model was fitted on the integrated profile (i.e., the heat [J] over time curve), resulting in an s-shaped curve considered as a good approximation for microbial growth^15^. Additionally, OD_595_ curves are hill-shaped as some microorganisms might undergo lysis. To neglect this effect in the analysis the OD_595_ values were considered to be constant once they had reached the maximum, which allowed an adequate modelling of the OD_595_ curve.

All those transformations enabled to compare directly all the curves generated by IMC, TDLAS and OD_595_.

#### Determination of Time to Detection (TtD)

Once the thresholds for CO_2_ (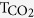), O_2_ (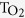), Heat (T_H_) and OD_595_ (T_OD_) were determined, each TDLAS, IMC and OD_595_ profile was described by λ, μ and X_max_ and the intersection with the respective threshold identified. For TDLAS and IMC a clear definition of lower (and upper) growth boundaries was needed to account for biological variation in growth and to determine a representative organism-related absolute TtD (see Fig. 2). Lower growth boundaries (B_low_) were defined by combining maximal λ with smallest μ and lowest X_max_ in the Gompertz model. The intersection of B_low_ and the respective threshold determined the absolute TtD that CO_2_, O_2_ or heat measurements needed to detect either *S. salivarius* or *B. subtilis* (Fig. 3). Upper boundaries (B_up_) were defined through combining minimal λ with highest μ and largest X_max_ in the Gompertz model. The definition of B_low_ and B_up_ imposes a measure for estimating total growth distribution. TtD for VI was defined as point where 100% of all inoculated samples turned turbid and is of qualitative nature.

## Additional Information

**How to cite this article**: Brueckner, D. *et al*. Comparison of Tunable Diode Laser Absorption Spectroscopy and Isothermal Micro-calorimetry for Non-invasive Detection of Microbial Growth in Media Fills. *Sci. Rep.* **6**, 27894; doi: 10.1038/srep27894 (2016).

## Figures and Tables

**Table 1:** Time to detection, lag phase, growth factor and reached X_max_ for *B. subtilis* and *S. salivarius*, related to methods under investigation are presented.

	TDLA 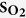 (min–max) ( 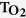 = 1.754%)	TDLA 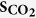 (min-max) ( 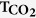 = 0.366%)	IMC_HEAT_ (min-max) (T_H_ = 0.416J)	OD_595_ [remaining values] (T_OD_ = 0.02)	Visual (all vials turbid)
***Bacillus subtilis***					
**n**	60	60	18	3	60
**TtD** (*time to detection*) [h] (intersection of B_low_ with threshold)	58.0 (36.7–57.0)	52.6 (35.7–54.4)	45.9 (34.4–47.2)	33.6 [28.3; 31.8]	45.0 (24.5–45.0)
**λ** (*lag phase*) [h]	39.85 (34.59–55.38)	45.65 (41.08–58.37)	40.58 (37.80–48.45)	36.40 (33.78–37.58)	n.a.
**μ** (*growth rate*) [% or J/h]	0.46 (0.35–0.61)	0.46 (0.38–0.58)	0.40 (0.30–0.63)	0.06 (0.05–0.06)	n.a.
**X**_**max**_ (*C*_max_*/Heat*) [% or J]	19.89 (19.35–20.93)	17.93 (17.13–18.69)	12.79 (11.11–13.98)	1.55 (1.44–1.57)	n.a.
***Streptococcus salivarius***					
**n**	57	57	22	3	57
**TtD** (***time to detection***) **[h]** (intersection of B_low_ with threshold)	ND (51.0–ND)	76.0 (45.2–70.7)	71.2 (44.3–69.3)	51.9 [40.5; 50.0]	61.0 (37.3–61.0)
**λ** (*lag phase*) [h]	34.74 (17.75–57.20)	48.14 (40.12–68.96)	46.67 (42.20–65.74)	50.78 (41.55–52.40)	n.a.
**μ** (*growth rate*)[ [% or J/h]	0.06 (0.017–0.1)	0.15 (0.05–0.33)	0.12 (0.08–0.20)	0.23 (0.08–0.57)	n.a.
**X**_**max**_ (*C*_max_*/heat*) [% or J]	2.12 (1.41–3.4)	2.22 (1.13–3.14)	2.37 (1.79–2.90)	1.25 (0.78–1.75)	n.a.

General TtD is expressed as number of hours where the lower growth boundary (B_low_) crosses 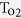, 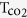 or T_H_. In brackets the TtD distribution of each single replicate. ND stands for not detected and is a direct measure for sensitivity as related to false negative results. Lag phase (λ), growth factor (μ) and maximal value reached (X_max_) are demonstrated in form of median and range (in brackets). Unit for λ is hours, for X_max_ percentage, whereby μ goes without unit. Parameters were received from applying the Gompertz model in R (“grofit” package) on each individual growth profile. Measured OD_595_ values are of descriptive nature and used as reference method to show meaningfulness of results obtained for TDLAS and IMC. As a substantial number of OD_595_ samples was investigated it would lead to misconceptions in statistics if used differently. The single number is the maximal value of all three OD_595_ measurement runs. In brackets the remaining values.

**Table 2:** Describes and compares advantages and drawback of TDLAS, IMC and visual inspection.

Advantages/Disadvantages	TDLAS	IMC	VI
Objective inspection (quantitative results)	+ +	+ +	− −
High throughput rate	+ +	+	− −
Efficient and automatic data collection	+ +	+ +	− −
Measurement Sensibility/Speed in detection	+	+	+ +
Non-invasive inspection	+ +	+ +	+ +

+ + fully applicable, + partly applicable, − partly not applicable, − − not applicable.
